# *Helicobacter pylori* Outer Membrane Protein 18 (Hp1125) Is Involved in Persistent Colonization by Evading Interferon-***γ*** Signaling

**DOI:** 10.1155/2015/571280

**Published:** 2015-04-06

**Authors:** Yuqun Shan, Xingxiao Lu, Yingnan Han, Xinpeng Li, Xiao Wang, Chunhong Shao, Lixiang Wang, Zhifang Liu, Wei Tang, Yundong Sun, Jihui Jia

**Affiliations:** ^1^Department of Microbiology, Key Laboratory for Experimental Teratology, Chinese Ministry of Education, School of Medicine, Shandong University, Jinan, Shandong 250012, China; ^2^Shandong Center for Disease Control and Prevention, Jinan 250010, China; ^3^Clinical Laboratory, Shandong Provincial Hospital Affiliated to Shandong University, Jinan, Shandong 250012, China; ^4^Institute of Pharmacology, School of Medicine, Shandong University, Jinan, Shandong 250012, China

## Abstract

Outer membrane proteins (OMPs) can induce an immune response. Omp18 (HP1125) of *H. pylori* is a powerful antigen that can induce significant interferon-*γ* (IFN-*γ*) levels. Previous studies have suggested that IFN-*γ* plays an important role in *H. pylori* clearance. However, *H. pylori* has multiple mechanisms to avoid host immune surveillance for persistent colonization. We generated an *omp18* mutant (*H. pylori* 26695 and *H. pylori* SS1) strain to examine whether Omp18 interacts with IFN-*γ* and is involved in *H. pylori* colonization. qRT-PCR revealed that IFN-*γ* induced Omp18 expression. qRT-PCR and western blot analysis revealed reduced expressions of virulence factors CagA and NapA in *H. pylori* 26695 with IFN-*γ* treatment, but they were induced in the Δ*omp18* strain. In C57BL/6 mice infected with *H. pylori* SS1 and the Δ*omp18* strain, the Δ*omp18* strain conferred defective colonization and activated a stronger inflammatory response. Signal transducer phosphorylation and transcription 1 (STAT1) activator was downregulated by the wild-type strain but not the Δ*omp18* strain in IFN-*γ*-treated macrophages. Furthermore, Δ*omp18* strain survival rates were poor in macrophages compared to the wild-type strain. We concluded that *H. pylori* Omp18 has an important function influencing IFN-*γ*-mediated immune response to participate in persistent colonization.

## 1. Introduction

*Helicobacter pylori* is the principal pathogenic factor in gastritis, peptic ulcer, and even gastric cancer and mucosa-associated lymphoid tumors [1, 2]. Almost half of the world's population has had an* H. pylori* infection, especially in China [3]. Without treatment,* H. pylori *colonizes in the stomach for the host's entire life [4]. Therefore,* H. pylori* has near-perfect niche adaptation and can avoid human immune responses [5, 6].

Most outer membrane proteins (OMPs) of bacteria are surface-exposed and therefore may be important in interfacing bacteria with the mammalian host and its defenses [7]. For example,* Pseudomonas aeruginosa* OprF can recognize IFN-*γ* and mount an effective countermeasure to immune activation by the host [8].* Francisella novicida* FopC plays a role in inhibiting the IFN-*γ*-mediated host immune defense [9].* H. pylori* contains an OMP family of approximately 33 genes [10]. Omp18 (HP1125), located on bacteria's outer membrane surfaces, is expressed by all known* H. pylori* strains and can react specifically with sera from all* H. pylori*-infected patients. Omp18 is habitually recognized by the immune system [11] and primes a T helper 1 cell (Th1) response from proliferating splenocytes by inducing IFN-*γ* production [12].

*H. pylori* infection is dominated by the Th1-type immune response [13, 14]. IFN-*γ* is a characteristic Th1 response cytokine [15], and IFN-*γ* activity, mediated by a CD4^+^ T-cell response to* H. pylori* infection, is essential for clearance [16, 17]. IFN-*γ* can induce nitric oxide (NO) production in macrophages by activating the transcription factor signal transducer and activator of transcription 1 (STAT1) [18], and NO is a key component of the innate immune system and an effective antimicrobial agent [19]. However,* H. pylori* can disrupt STAT1-mediated IFN-*γ*-induced signal transduction in epithelial cells [20]. Moreover, when* H. pylori* is exposed to IFN-*γ*; the main virulence factor cytotoxin-associated gene A (CagA) is downregulated [21], which is beneficial for persistent colonization [22]. Thus,* H. pylori* may actively respond to altered IFN-*γ* levels for persistent colonization.

Considering Omp18's importance to* H. pylori*, we constructed an* omp18* mutant strain to study this protein's contribution to* H. pylori*'s persistent colonization. Omp18 helped to inhibit IFN-*γ*-mediated* H. pylori* virulence factors and host immune response, thereby promoting colonization.

## 2. Materials and Methods

### 2.1. Bacteria and Culture Conditions

*H. pylori* 26695 and the SS1 strain were kindly provided by Dr. Zhang Jianzhong (Chinese Disease Control and Prevention Center). The bacteria were revived from frozen stocks and grown on Skirrow agar with 5% (v/v) sheep's blood under microaerobic conditions (5% O_2_, 10% CO_2_, and 85% N_2_) at 37°C. The liquid culture media for* H. pylori* consisted of Brucella broth containing 10% fetal bovine serum for incubation in a microaerobic environment at 37°C on a shaker set at 120 rpm. For* Δomp18* isogenic mutants, kanamycin (10 mg/mL, Sigma-Aldrich, St. Louis, MO) was supplemented in solid and liquid medium. We supplemented 10 mL aliquots of liquid overnight-cultured* H. pylori* 26695 and* Δomp18* isogenic mutants with IFN-*γ* concentrations (Sigma-Aldrich) to examine the effects on* omp18, cagA, and napA*.

### 2.2. Construction of the* omp18 *Mutant Strain

The* omp18* mutant strains for* H. pylori* 26695 and SS1 were constructed as described [23]. Plasmids pILL570 and pUC18K2 were kindly provided by Dr. Agnes Labigne (Département de Microbiologie, Unité de Pathogénie Bactérienne des Muqueuses, Institut Pasteur, Paris). The mutant strains were constructed as follows: fragment 1 containing the 5′ region of the* omp18* gene flanked by* Cla*I and* Eco*RI restriction sites was amplified by PCR with the first pair of primers (*omp18*-1 and* omp18*-2). Fragment 2, containing the 3′ region of* omp18* flanked by* Bam*HI and* Pst*I restriction sites, was amplified by PCR with the second pair of primers (*omp18*-3 and* omp18*-4).* H. pylori* 26695 and SS1 genomic DNA were used as the template, and the primers are in Table 1. Following PCR amplification, fragment 1 was digested by* Cla*I and* Eco*RI, and fragment 2 was digested by* Bam*HI and* Pst*I. We obtained the nonpolar kanamycin cassette from pUC18K2 by* Eco*RI and* Bam*HI digestion. Then, we ligated the resulting 3 fragments with the* Cla*I- and* Pst*I-digested vector pILL570 by T4 ligase (Fermentas), generating the plasmid pILL570-*omp18*, in which about a 200-bp* omp18* deletion was replaced by the kanamycin cassette. Finally,* H. pylori* 26695 and SS1 were electrotransformed with the plasmid pILL570-*omp18*, and we selected kanamycin-resistant (Kanr) recombinants. The* omp18* mutation in the Kanr recombinant was verified by PCR with the primers for* omp18*-1 and* omp18*.

### 2.3. Care and Infection of Experimental Mice, Gastric Tissue Preparation, and Assessment of Colonization and Histopathology

The use of animals in this experiment was approved by the Ethics Committee of Shandong University School of Medicine (number 001 in 2011 for Animal Ethics Approval), and all efforts were made to minimize the mice's suffering. We obtained 80 6-week-old, female, specific-pathogen-free (SPF) C57BL/6 mice from Zhejiang University that were bred at the Shandong University pathogen-free facility. We allowed the mice to adapt to their new environment for 2 weeks before experimentation. We housed them in individual ventilated cages and, at 8 weeks old, divided them into 2 groups (*n* = 40/group) for inoculation by oral gavage twice over 3 days with 100 *μ*L* H. pylori* SS1 (~10^8^ colony-forming units [cfu] mL^−1^) or 100 *μ*L* H. pylori* SS1 Omp18 isogenic mutant (~10^8^ cfu mL^−1^).

Five mice from each group were euthanized by CO_2_ asphyxiation at 2, 4, 6, and 8 weeks after inoculation. We retrieved and cleaned their stomachs and removed the forestomach. We opened the remaining piece containing the corpus and antrum along the lesser curvature and spread it out in the form of a trapeze. We then dissected the tissue longitudinally (i.e., from the forestomach/corpus junction down to the antrum/duodenum junction) into 3 equal, parallel pieces with nearly identical antral and corpus tissue proportions. To quantitatively assess* H. pylori* colonization, we transferred one section from each stomach to a tube containing Brucella broth and homogenized them. We placed serial dilutions on horse blood plates to determine bacterial loads. We homogenized one section from each stomach for ELISA, fixed the last section in 10% neutralized buffered formalin, and then embedded them in paraffin. Paraffin blocks were sectioned and stained with haematoxylin and eosin for histopathological evaluation or with Steiner's modified silver stain to grade bacterial load. Polymorphonuclear and mononuclear cells in the antrum and body were graded as described [24]: 0, none; 1, some infiltrates; 2, mild infiltrates (few aggregates in submucosa and mucosa); 3, moderate infiltrates (several aggregates in submucosa and mucosa); 4, marked infiltrates (many large aggregates in submucosa and mucosa); 5, nearly the entire mucosa contained a dense infiltrate; and 6, the entire mucosa contained a dense infiltrate.

### 2.4. Cell Culture and Infection Conditions

We maintained murine macrophage RAW264.7 cells (from BOSTER, Wuhan, China) in DMEM (Gibco, USA) supplemented with 10% (v/v) fetal bovine serum (FBS). The human gastric epithelial cell line AGS, obtained from the cell repository for Academia Sinica (Shanghai), was grown in F12 (Gibco, USA) supplemented with 10% (v/v) fetal bovine serum (FBS). All cell lines were incubated in a humidified atmosphere containing 5% CO_2_ at 37°C without antibiotics. Before infection,* H. pylori* 26695 bacteria was washed and resuspended in a total volume of 0.05 mL antibiotic-free tissue culture medium. The cell lines were infected with* H. pylori* 26695 at a multiplicity of infection (MOI) of 1 : 100 in antibiotic-free medium and incubated in a humidified atmosphere containing 5% CO_2_ at 37°C. After infection, we rinsed the cell monolayer and added medium alone or medium containing IFN-*γ* (50 pg/mL) to the remaining adherent cells. We confirmed bacteria viability after the experiments concluded by visualizing their motility under light microscopy.

### 2.5. ELISA

AGS cells were seeded at 1 × 10^5^/well in 24-well plates and incubated in 95% air and 5% CO_2_ humidified air for 24 h at 37°C. After infection with* H. pylori* strain with or without IFN-*γ* (50 pg/mL) for 15 h, the supernatant was harvested and stored at −80°C after aspiration. We detected interleukin-8 (IL-8) secreted by AGS cells after stimulation for 15 h using a human IL-8 ELISA kit (NeoBioscience, China). The homogenized stomach was centrifuged at 20,000 g for 10 min at 4°C to precipitate the insoluble cellular debris, and the supernatant was stored at −80°C. IFN-*γ*, macrophage inflammatory protein 2 (MIP-2), and IL-12p70 protein levels in supernatants were assayed in duplicate with mouse ELISA kits specific for IFN-*γ* (eBioscience, San Diego, CA), MIP-2 (Cusabio Biotech, China), and IL-12p70 (NeoBioscience, China), respectively.

### 2.6. Quantitative Real-Time PCR

To determine the mRNA expressions of* Omp18*,* NapA*, and* CagA* with or without IFN-*γ* treatment, we inoculated* H. pylori* 26695 and 26695* Δomp18* isogenic mutants into liquid bacterial cultures with preliminary OD_600_ 0.05 and harvested them at different times. We extracted the total bacterial RNA using TRIzol (Invitrogen). Then, we obtained cDNA using the Revert Aid First Strand cDNA Synthesis Kit (Fermentas). Quantitative RT-PCR amplification involved the ABI Prism 7000 Sequence Detection System (Applied Biosystems, Carlsbad, CA) with one cycle at 95°C for 10 s and 40 cycles at 95°C for 5 s and 60°C for 31 s. Each reaction mixture contained 10 *μ*L SYBR Premic Ex TaqTM (Takara, Otsu, Shiga, Japan) and 0.4 *μ*L ROX Reference Dye (Takara) added to each 20 *μ*L PCR reaction mixture. We performed a melting curve analysis for each PCR reaction to ensure the amplified product's purity. The data were normalized to 16sRNA (*H. pylori*) expression in each sample, with 3 biological replicates performed. We calculated the relative gene expression using the 2^−(ΔΔCt)^ method. PCR amplification involved the primers listed in Table 1.

### 2.7. Western Blot Analysis

To detect the* NapA* and* CagA* protein expressions with or without IFN-*γ* treatment, we collected the liquid bacterial culture of* H. pylori* 26695 and 26695* Δomp18* isogenic mutants at 8 h. Bacterial cell lysates were prepared as described [25]. To determine phosphotyrosine STAT1 protein expression, we seeded macrophages at 2 × 10^6^/well in flat-bottomed 6-well microplates for 24 h and then infected them with* H. pylori 26695* with or without IFN-*γ* (50 pg/mL) for 6 h. Cells were washed twice with ice-cold phosphate buffered saline (PBS) and lysed in RIPA buffer (Beyotime Biotechnology, China) with 1% PMSF. We spun down the lysates and collected the supernatant. We used the Bradford method to determine protein concentration. Approximately 25–30 *μ*g protein for each sample was loaded and separated by SDS-PAGE. They were then probed with specific antibodies against NapA (obtained from our lab) [25], STAT1 (Cell Signaling Technology, #5375), and CagA (Abcam, ab90490), followed by anti-mouse or rabbit horseradish peroxidase-conjugated IgG. They were then developed with the enhanced chemiluminescence method. *β*-Actin was a loading control, and each experiment was repeated 3 times.

### 2.8. Griess Assay of Nitrite Concentration

Macrophages were seeded at 2 × 10^6^/well in six-well plates and incubated in 95% air and 5% CO_2_ humidified air for 24 h at 37°C. After infection with* H. pylori* 26695 with or without IFN-*γ* (50 pg/mL) for 3 h, we collected the supernatant and stored it at −80°C. We estimated the nitrite content in infected macrophages and gastric tissue supernatant by a colorimetric assay based on the Griess reaction [26]. Briefly, 50 *μ*L supernatant was mixed with 50 *μ*L Griess reagent I and II (Beyotime, China). We measured the absorbance at 540 nm (Bio-Rad) and determined nitrite concentration by extrapolation using a NaNO_2_ standard curve (1–100 mM).

### 2.9. Survival of Wild-Type and* Δomp18 H. pylori* Exposed to Sodium Nitroprusside (SNP)

This experiment was performed as we previously described [27], with some modifications. We added overnight-cultured* H. pylori* (OD_600_ ≈ 0.8) to SNP (8 mM) and added* H. pylori* suspensions at 0, 2, 4, 6, and 12 h to SA plates with 5% (v/v) sheep's blood and then incubated them under microaerobic conditions (5% O_2_, 10% CO_2_, and 85% N_2_) at 37°C for 3 to 4 days before viability assessment. To assess viability at each time, we determined the number of colony-forming units by plating serial dilutions of cultures in duplicate on Skirrow agar plates with 5% (v/v) sheep's blood. Each assay was replicated at least 3 times.

### 2.10. Confocal Microscopy

To determine shape and survival ability of* H. pylori* exposed to SNP, we stained* H. pylori* (OD_600_ ≈ 0.8) using LIVE/DEAD BacLight Bacterial Viability kits (Molecular Probes, Invitrogen, USA) and then performed confocal microscopy as described [25]. SYTO-9 is a green fluorescent membrane-permeant dye that labels all bacteria by staining nucleic acid, whereas PI is a red-fluorescent membrane-impermeant dye that labels only bacteria with damaged membranes.

### 2.11. Intracellular Bacterial Survival Assay

Survival of wild-type and* Δomp18 H. pylori* in macrophages was demonstrated as previously described [28], with some modifications. Cells were seeded at 5 × 10^5^/well in 24-well plates and incubated in 37°C, 95% air, and 5% CO_2_ humidified air conditions for 24 h. Macrophages were infected at 100 MOI with chilled wild-type or* Δomp18 H. pylori* and then incubated in 37°C, 95% air, and 5% CO_2_ humidified air conditions. After 1 h, infected monolayers were washed once with PBS and then incubated in 500 *μ*L DMEM containing 10% (v/v) FBS and 100 *μ*g gentamicin/mL for 1 hr to kill extracellular bacteria but not macrophages. Infected cells were then lysed at different times (2, 6, and 24 h). To lyse macrophage monolayers and release* H. pylori*, we added 500 *μ*L sterile water to each well. Finally, we determined the number of viable bacteria in macrophage lysates by plating serial dilutions on solid plates.

### 2.12. Statistical Analysis

Data are presented as means ± SEM. Statistical significance was determined by unpaired Student's *t* test, and the *P* values were corrected by the Sidak-Bonferroni method for multiple comparisons. *P* < 0.05 was considered statistically significant. Results were analyzed using a Graphpad Prism (Graphpad Software Inc., La Jolla, CA, USA).

## 3. Results

### 3.1. IFN-*γ* Induced Higher Expression of* H. pylori *26695 Omp18

**Figure 1:**
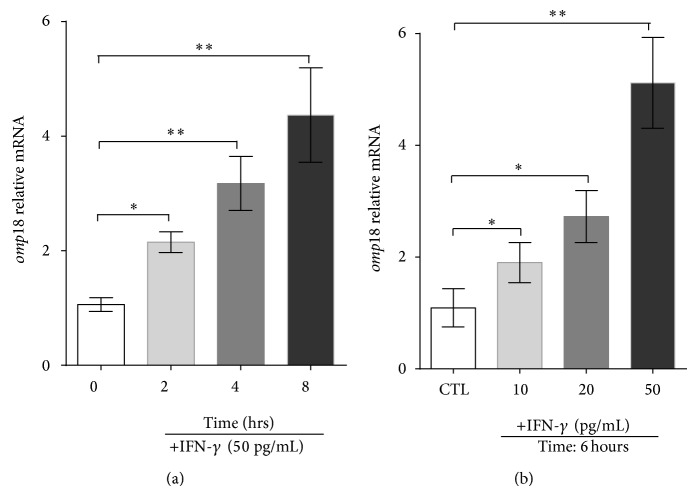
Interferon *γ* (IFN-*γ*) induces high* omp18* expression in* Helicobacter pylori 26695*. Quantitative RT-PCR analysis of* Omp18* mRNA expression with (a) exposure to different concentrations of IFN-*γ* and (b) exposure to IFN-*γ* (50 pg/mL) at different times. Quantitative RT-PCR data were standardized to that of 16s rRNA. Data are means ± SEM from 3 independent experiments. ^∗^ *P* < 0.05, ^∗∗^ *P* < 0.01.

Because IFN-*γ* is a predominant component of the anti-*H. pylori* protective immune response [16, 17] and because Omp18 induces IFN-*γ* production [12], we wondered whether IFN-*γ* could affect Omp18 expression, and our results displayed that IFN-*γ* induced higher expression of* H. pylori* 26695 Omp18 by dose (Figure 1(a)) and time (Figure 1(b)).

### 3.2. IFN-*γ* Reduces the Expressions of* H. pylori* 26695 Virulence Factors CagA and NapA

**Figure 2:**
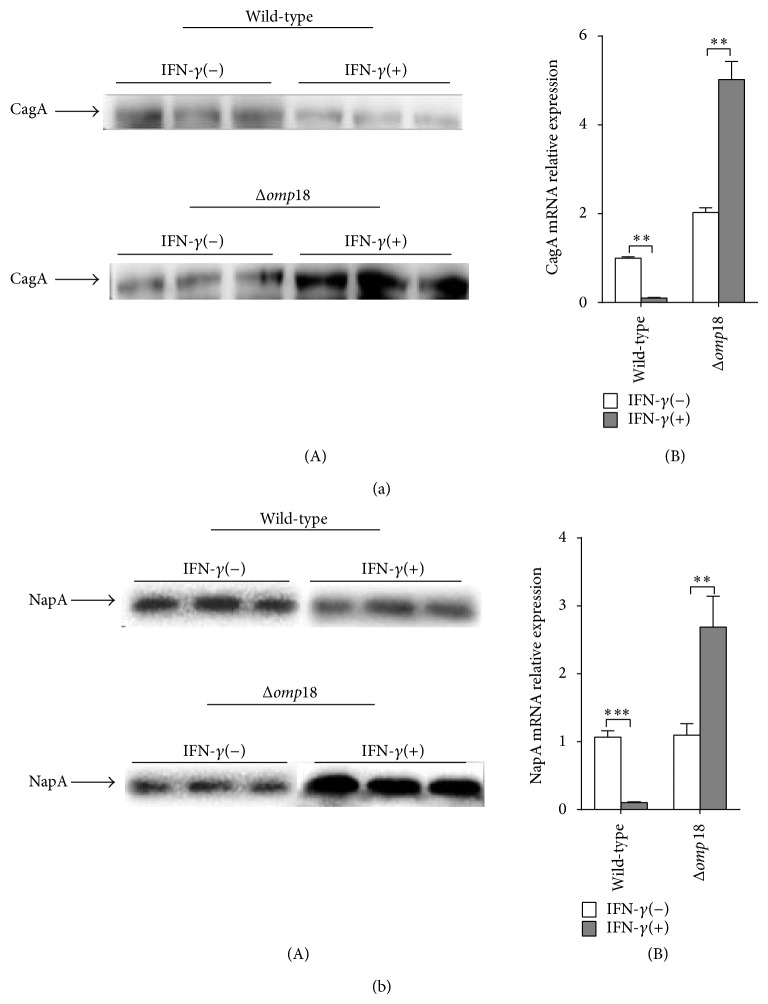
IFN-*γ* reduces wild-type* H. pylori* virulence factor expressions. Wild-type and* Δomp18 H. pylori* were cultured to OD_600_ ≈ 0.8 and then exposed or not to IFN-*γ* (50 pg/mL) for 6 h. Western blot and qRT-PCR analysis of protein (A) and mRNA (B) levels of CagA (a) and NapA (b). Quantitative RT-PCR data were standardized to that of 16s rRNA. Data are means ± SEM from 3 independent experiments. ^∗∗^ *P* < 0.01, ^∗∗∗^ *P* < 0.001.

CagA and NapA are important virulence factors involved in the* H. pylori* pathogenic process [1, 2]. Therefore, we sought to determine their expression when* H. pylori* was exposed to IFN-*γ*. CagA and NapA were downregulated in wild-type* H. pylori* 26695 exposed to IFN-*γ* but could not be reduced in the* Δomp18* strain with IFN-*γ* treatment (Figure 2).

### 3.3. *Δomp18 H. pylori* Shows Defective Colonization in Mice's Gastric Systems

**Figure 3:**
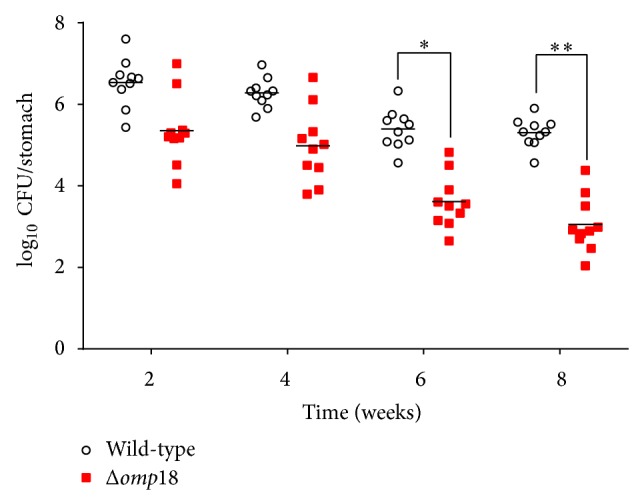
*Δomp18 H. pylori* shows defective colonization in mice gastric systems. C57BL/6 mice (*n* = 10/group, 6–8 weeks old) were infected with* H. pylori* SS1 and* Δomp18 H. pylori* SS1 (10^9^/mL). ^∗^ *P* < 0.05, ^∗∗^ *P* < 0.01. CFU: colony formation unit.

Previous studies reported that several OMPs participate in* H. pylori* colonization [29]. To evaluate the difference in colonization efficiency between* H. pylori* SS1 and the* Δomp18 H. pylori* SS1 strain, we inoculated mice with these two kinds of bacteria and euthanized them from 2 to 8 weeks after inoculation. Compared to* H. pylori* SS1, the* Δomp18* strain showed gradually decreased colonization from weeks 2 to 8 and especially weeks 6 and 8 in C57BL/6 mice (Figure 3).

### 3.4. Omp18 Isogenic Mutant Strain's Effect on Infection Severity in Mice

**Figure 4:**
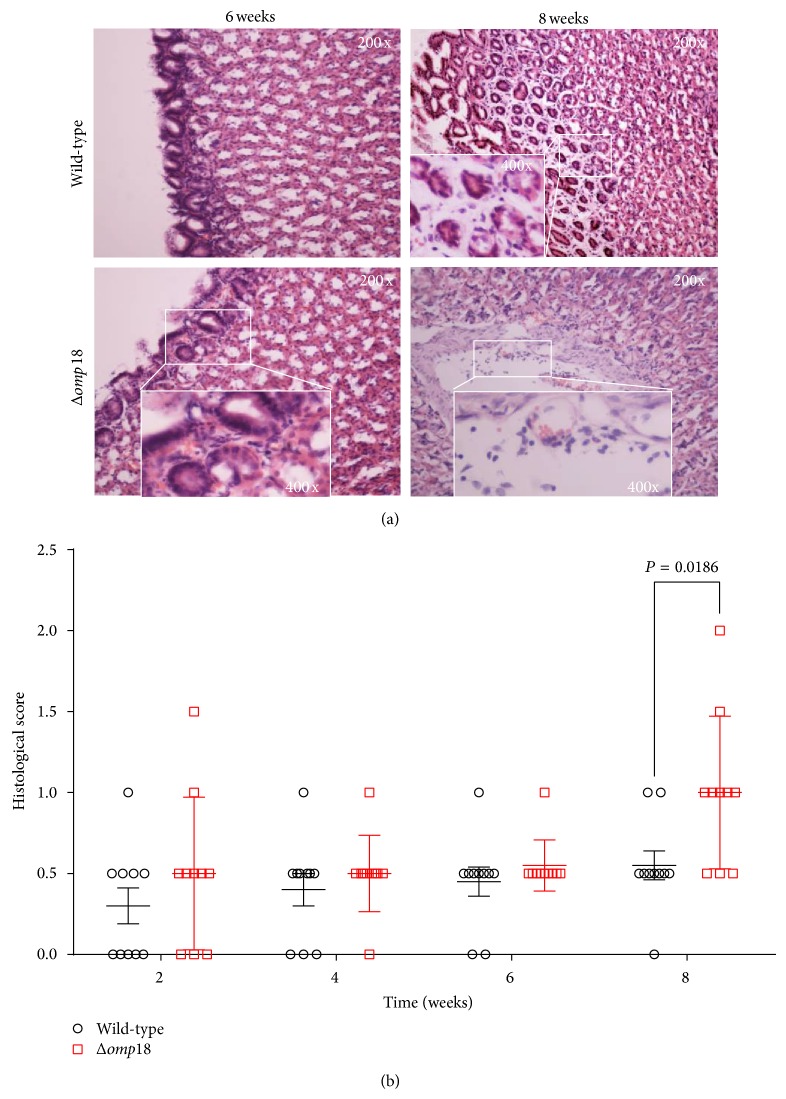
*Δomp18 H. pylori* SS1 infection induces inflammation response in C57BL/6 mice. (a) Histology of antrum from C57BL/6 mice infected with* H. pylori* SS1 or* Δomp18 H. pylori* SS1 at weeks 6 and 8. All sections were stained with hematoxylin and eosin. Magnification: 200x, 400x. Areas of lymphocytic inflammation were marked by rectangles. (b) Histopathology scores for gastric tissues from C57BL/6 mice (*n* = 10/group, 6–8 weeks old).

Because the inflammation score is likely affected by* H. pylori* density, we measured the inflammation score in relation to* H. pylori* density (score/log_10_ cfu). Histologic changes in the gastric mucosa infected by* H. pylori* SS1 were very mild or undetected from 2 to 6 weeks after inoculation. However, gastric mucosa infected with the Omp18 isogenic mutant strain showed histologic changes at 8 weeks of inoculation, and the inflammation score was higher than that for wild-type-infected mice (Figures 4(a) and 4(b)).* Δomp18*-infected mice showed increased neutrophil infiltration and severe gastric tissue damage, with the greatest damage at week 8 (*P* = 0.0186).

### 3.5. *Δomp18 H. pylori* Infection Induces More Cytokine and Chemokine Production

**Figure 5:**
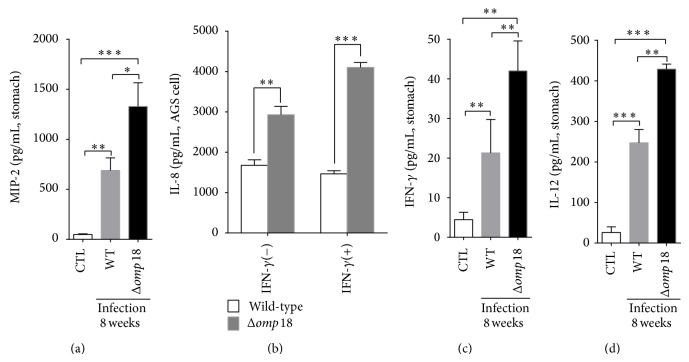
*Δomp18 H. pylori* infection increased cytokine and chemokine production. (a), (c), and (d) Analysis of MIP-2, IFN-*γ*, and IL-12 protein expressions in gastric tissues from C57BL/6 mice infected by* H. pylori* SS1 or* Δomp18 H. pylori* SS1 at week 8. (b) Analysis of IL-8 secretion from AGS cells infected by* H. pylori* 26695 or* Δomp18 H. pylori 26695* with or without IFN-*γ* (50 pg/mL). Data are means ± SEM from 3 independent experiments. ^∗^ *P* < 0.05, ^∗∗^ *P* < 0.01, and ^∗∗∗^ *P* < 0.001.

At 8 weeks after inoculation, compared with wild-type infection,* Δomp18* infection increased MIP-2, IFN-*γ*, and IL-12p70 production in gastric tissues (Figures 5(a), 5(c), and 5(d)). More IL-8 expression was induced in human gastric cancer AGS cells infected with* Δomp18 H. pylori* SSI with or without IFN-*γ* incubation (Figure 5(b)).

### 3.6. *Δomp18 H. pylori* Infection Induces More NO Production

**Figure 6:**
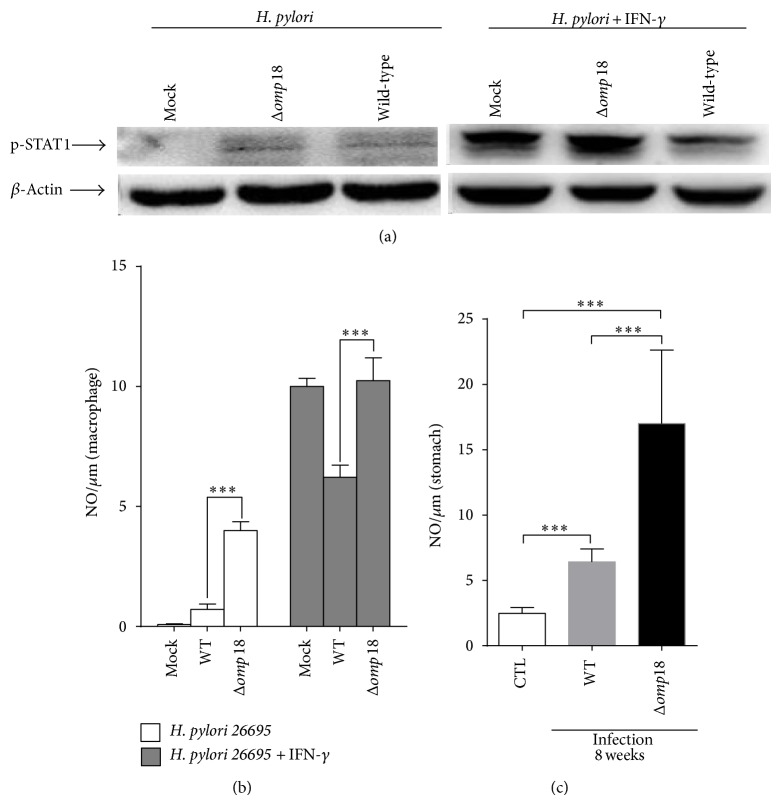
*Δomp18 H. pylori* induces more nitric oxide (NO) production in macrophages and mice stomachs. The murine macrophage RAW264.7 was used in this experiment and was infected by* H. pylori* 26695 or* Δomp18 H. pylori 26695* with or without IFN-*γ* (50 pg/mL). (a) Western blot analysis of phosphorylated signal transducer and regulator of transcription 1 (STAT1) protein expression in murine macrophage RAW264.7 cells. Actin was a loading control. (b) Analysis of NO secretion from murine macrophage RAW264.7 cells by ELISA. (c) Analysis of NO secretion in gastric tissues from C57BL/6 mice infected by* H. pylori* SS1 and* Δomp18 H. pylori* SS1 at week 8 by ELISA. *n* = 10/group, 6–8 weeks old. ^∗∗∗^ *P* < 0.01. Data are means ± SEM from 3 independent experiments.

Phosphorylated STAT1 is associated with NO production from macrophages and could be induced by IFN-*γ* [18]. With IFN-*γ* treatment, STAT1 phosphorylation in macrophages was inhibited with wild-type but not* Δomp18 H. pylori* (Figure 6(a)). Also,* Δomp18* infection increased NO production from macrophages with or without IFN-*γ* (Figure 6(b)). At 8 weeks after inoculation, compared with wild-type infection,* Δomp18 H. pylori* infection induced more NO secretion (Figure 6(c)).

### 3.7. Omp18 Is Involved in* H. pylori* 26695 Survival with Oxidative Stress and Antiphagocytosis

**Figure 7:**
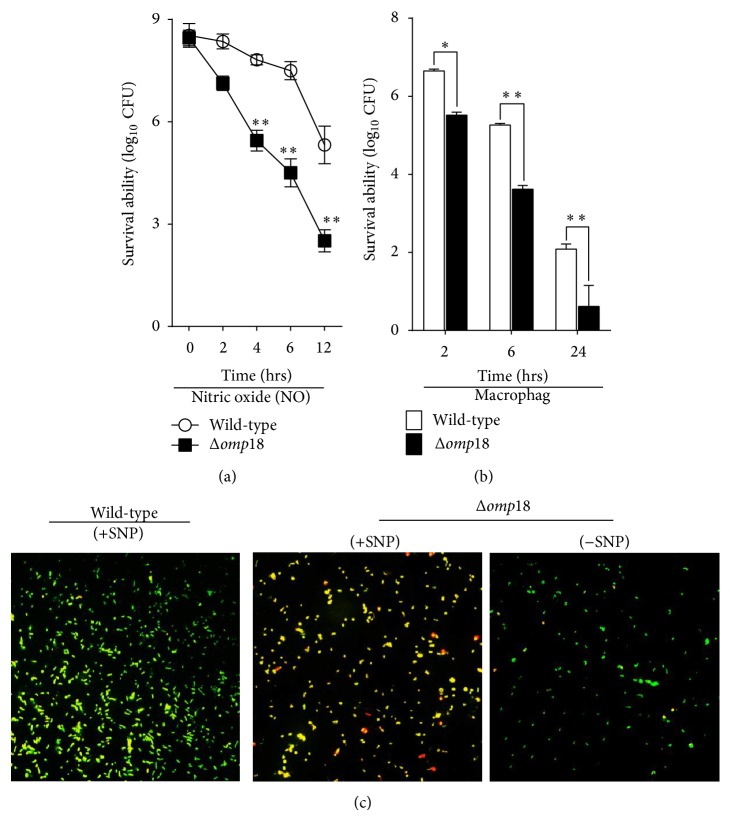
Omp18 is involved in* H. pylori* survival with NO oxidative stress and in macrophages. (a) Survival of wild-type and* Δomp18 H. pylori* with exposure to NO oxidative stress (simulation with sodium nitroprusside, SNP). CFU: colony formation unit. (b) Survival of wild-type and* Δomp18 H. pylori* in macrophages. Data are means ± SEM from 3 independent experiments. (c) SNP treatment for 6 h induces coccoid transformation and death of* Δomp18* bacteria, and* Δomp18 H. pylori* without SNP treatment was a negative control. Confocal microscopy of cells stained with membrane-permeant SYTO-9 (green) and membrane-impermeant PI (red). Data are representative of 3 independent experiments. ^∗^ *P* < 0.05, ^∗∗^ *P* < 0.01.

*H. pylori* colonization is inevitably attacked by reactive oxygen species and eliminated by macrophages. With SNP as a simulation for NO,* Δomp18* bacteria survival rates decreased sharply compared to wild-type* H. pylori* (Figure 7(a)), and most bacteria transformed from normal helical bacillary morphology to a coccoid morphology (Figure 7(c)). The* Δomp18* strain also showed weakened survival rates in macrophages (Figure 7(b)).

## 4. Discussion

*H. pylori* can colonize the human stomach in childhood, and, without treatment, it can persist throughout life [30].* H. pylori* can successfully cope with innate immune responses and continuous attack by the adaptive immune response [6] and has evolved complex mechanisms to escape immune reactions. For example,* H. pylori* lipopolysaccharide is not well recognized by Toll-like receptor 4 (TLR4) [31], and flagellin is a poor TLR5 stimulator [32]. Also, vacuolating cytotoxin A interferes with antigen processing in B cells [33]. In this report, we focused on* H. pylori* Omp18's role in avoiding IFN-*γ* signaling to achieve persistent colonization in an experimental mouse model. First, the expression of Omp18 was induced by IFN-*γ in vitro*, and the expression of virulence factors CagA and NapA was reduced in* H. pylori* 26695 with IFN-*γ* treatment but induced in the* Δomp18* strain. Second, in C57BL/6 mice infected with* H. pylori* SS1 and the* Δomp18* strain, the* Δomp18* strain conferred defective colonization and activated a stronger inflammation response. Third, STAT1 phosphorylation is downregulated by the wild-type but not* Δomp18* strain in IFN-*γ*-treated macrophages. Furthermore,* Δomp18* strain survival rates are poor in macrophages compared to the wild-type strain. Our data showed that Omp18 in* H. pylori* may actively sense altered IFN-*γ* levels and respond to avoid the IFN-*γ*-mediated immune defense(s) involved in* H. pylori* colonization.

Although most* H. pylori* cells are found in the mucus layer covering the gastric epithelium [34], some bacteria are even found in deeper tissues or intracellular locations [35, 36]. IFN-*γ* has an important function for clearing* H. pylori* [16]. Outer membrane protein reportedly evades IFN-*γ*-mediated host immune defenses for several intracellular bacteria [9, 37]. Several OMPs are also known to be involved in* H. pylori* adhesion, such as BabA, AlpA (HopC), AlpB (HopB), and HopZ [29, 38–40]. Omp18 is a peptidoglycan-associated lipoprotein (PAL) precursor, and the previous study saw it as a major antigenic molecule [11]. In this study, the increased Omp18 expression induced by IFN-*γ* suggested that Omp18 may be involved in* H. pylori*'s adaptation to the host immune response. Correspondingly, the next set of experiments confirmed the conclusion.* Δomp18 H. pylori* conferred defective colonization in C57BL/6 mice's gastric systems with relatively severe inflammatory responses and increased production of the proinflammatory cytokine MIP-2 in mouse gastric tissues, as well as IL-8 in AGS cells. IL-8 (CXCL-8) and its functional murine counterpart MIP-2 induce neutrophil attraction, activation, and transendothelial migration. IL-8 is strongly induced in human gastric cancer cells, such as the commonly used AGS cell line on coculture with* H. pylori*, and this signal is generally assumed to initiate the acute (neutrophil-dominated) inflammation in the early stages of* H. pylori* infection [41]. More IFN-*γ* was also induced in C57BL/6 gastric tissues infected by the* Δomp18* strain, combined with increased IL-12p70 expression, which is involved in the differentiation of naive T cells into Th1 cells [42]. A stronger Th1 immune response may have been induced in* Δomp18*-rather than wild-type-infected C57BL/6 mice. The relatively strong inflammation and immune responses induced by* Δomp18* infection in C57BL/6 mice's gastric systems may contribute to its defective colonization and attribute to the high expressions of virulence factors CagA and NapA in the* Δomp18* strain induced by IFN-*γ*.

CagA and NapA, two important* H. pylori* virulence factors, can activate a strong innate and adaptive immune response in the host. CagA induces IL-8 expression in gastric epithelial cells [43], and NapA promotes Th1 immune responses [44]. To avoid being cleared by the host immune system,* H. pylori* must downregulate virulence genes' expressions. For example, after persistent colonization, some of the* H. pylori* population may delete their Cag genes [22]. In previous studies, we demonstrated that* H. pylori* CagA is suppressed by IFN-*γ* treatment [21]. In this study, NapA expression was also suppressed by IFN-*γ*. Conversely, these two virulence factors were upregulated in Δ*omp18 H. pylori* exposed to IFN-*γ*, so* Δomp18 H. pylori* infection may induce a more serious immune response in the host than wild-type* H. pylori*. This represents a key factor leading to their weak colonization in C57BL/6 mice. Because IFN-*γ* can induce high* H. pylori* Omp18 expression and because virulence factors CagA and NapA were downregulated by IFN-*γ* in wild-type rather than* Δomp18 H. pylori*, our results indicate that the expressions of virulence factors CagA and NapA of* H. pylori* exposed to IFN-*γ* may be modulated by Omp18. Similiarly, IFN-*γ* reportedly binds to an outer membrane protein in* Pseudomonas aeruginosa*, OprF, resulting in the expression of a quorum-sensing dependentvirulence determinant, the PA-I lectin [8], and sigma E activity is regulated by OMP expressions in* E. coli* [45].

NO is a key component of the innate immune system and an effective antimicrobial agent [19]. However,* H. pylori* has evolved countermeasures against it. For example,* H. pylori* can disrupt STAT1-mediated IFN-*γ*-induced signal transduction in epithelial cells [20], and macrophage Arg2 induced by* H. pylori* inhibits inducible NO synthase translation, NO production, and bacteria killing* in vitro* [46]. We found that wild-type but not* Δomp18 H. pylori* could disrupt STAT1-mediated IFN-*γ*-induced signal transduction in macrophages. Moreover,* omp18* mutant-infected macrophages produced greater NO activity in the supernatant, and more NO was produced from the gastric systems of C57BL/6 mice infected with the* omp18* mutant strain, which was attributed to the invalid STAT1 disruption and associated with stronger immune response activated by* Δomp18* infection. Furthermore,* Δomp18 H. pylori* survival rates were weakened compared to the wild-type with NO exposure. Most* Δomp18* strains transformed their normal helical bacillary features to coccoid features. Omp18 has an important function in helping* H. pylori* avoid NO oxidative stress. Additionally,* Δomp18 H. pylori* showed reduced survival rates in macrophages, another reason why* Δomp18* could not effectively colonize C57BL/6 mice's gastric tissues.

## 5. Conclusions

Omp18 is involved in* H. pylori* persistent colonization.* H. pylori* may actively sense altered IFN-*γ* levels by Omp18 and respond by optimizing the virulence phenotype to avoid inducing a strong immune response, thereby guaranteeing persistent colonization. Omp18 is involved in* H. pylori* surviving NO oxidative stress and antiphagocytosis. Our studies provide new insight into* H. pylori*'s immune evasion.

## Figures and Tables

**Table 1:** Primers used in this study.

Primers used to construct *Δomp18* strain	
*omp18*-1: 5′-CCATCGATAACAAACGCTCTTTGGCTTC-3′	*omp18*-2: 5′-CGGAATTCGGCAATACCGATGAATTTGG-3′
*omp18*-3: 5′-CGGGATCCATGAAGAGATCTTCTGTATTTAG-3′	*omp18*-4: 5′-AAAACTGCAGTTACTTCATTAATTTGACATCC-3′

Primers for RT-PCR	

*napA*F: 5′-TGAAGAGTTTGCGGACAT-3′	*napA*R: 5′-R AGAGTGGAAGCTCGTTTT-3′
*cagA*F: 5′-AGCAAAAAGCGACCTTGAA-3′	*cagA*R: 5′-AGCCAATTGCTCCTTTGAGA-3′
*omp18*F: 5′-TGCTTTTGGAAGGCAATACC-3′	*omp18*R: 5′-CATTTGGGTTTGGTTTCACC-3′
*16SrRNA*F: 5′-GCTCTTTACGCCCAGTGATTC-3′	*16SrRNA*R: 5′-GCGTGGAGGATGAAGGTTTT-3′

F: forward primer; R: reversed primer.

Underlined letters indicate nucleotides added at the 5′ end to create a restriction site.

Restriction sites for *Cla*I (*omp18*-1), *Eco*RI (*omp18*-2), *Bam*HI (*omp18*-3), and *Pst*I (*omp18*-4) are underlined letters.
